# *N*-[4-(Propyl­sulfamo­yl)phen­yl]acetamide

**DOI:** 10.1107/S1600536811055528

**Published:** 2012-01-07

**Authors:** Saba Ahmad, Muhammad Akhyar Farrukh, Fahim Ashraf Qureshi, Islam Ullah Khan, Mehmet Akkurt

**Affiliations:** a Department of Chemistry, Government College University, Lahore 54000, Pakistan; b Department of Physics, Faculty of Sciences, Erciyes University, 38039 Kayseri, Turkey

## Abstract

In the title compound, C_11_H_16_N_2_O_3_S, the S atom has a distorted tetra­hedral geometry [maximum deviation: O—S—O = 119.48 (15)°]. The dihedral angles between the benzene ring and its propyl­sulfonamide and methyl­amide substituents are 71.8 (2) and 5.8 (1)°, respectively. In the crystal, mol­ecules are linked by N_m_—H⋯O_s_ (m = methyl­amide and s = sulfonamide) hydrogen bonds, forming *C*(8) chains along the *a* axis. The two mol­ecule chains are connected by N—H⋯O hydrogen bonds, generating *R*_3_^2^(18) rings. The crystal packing is further stabilized by weak inter­molecular C—H⋯O hydrogen bonds.

## Related literature

For background to sulfonamides, see: Adams (2001 ▶); Ahrens (1996 ▶); Betts *et al.* (2003 ▶); Faryal *et al.* (2011 ▶); Mayers (2009 ▶); Root (1999 ▶). For related structures, see: Faryal *et al.* (2011 ▶); Ahmad *et al.* (2011*a* ▶,*b* ▶). For computation of ring patterns formed by hydrogen bonds in crystal structures, see: Etter *et al.* (1990 ▶); Bernstein *et al.* (1995 ▶); Motherwell *et al.* (1999 ▶).
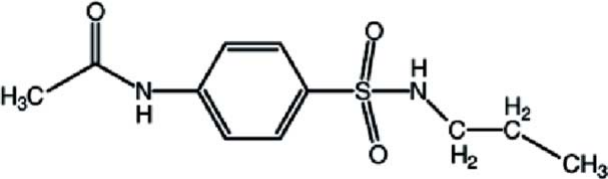


## Experimental

### 

#### Crystal data

C_11_H_16_N_2_O_3_S
*M* *_r_* = 256.33
Orthorhombic, 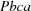

*a* = 8.7791 (6) Å
*b* = 14.1747 (11) Å
*c* = 20.1577 (14) Å
*V* = 2508.5 (3) Å^3^
*Z* = 8
Mo *K*α radiation
μ = 0.26 mm^−1^
*T* = 296 K
0.13 × 0.12 × 0.10 mm

#### Data collection

Bruker APEXII CCD diffractometer
22013 measured reflections
3103 independent reflections
1579 reflections with *I* > 2σ(*I*)
*R*_int_ = 0.077

#### Refinement

*R*[*F* ^2^ > 2σ(*F* ^2^)] = 0.057
*wR*(*F* ^2^) = 0.173
*S* = 1.01
3103 reflections
164 parameters
2 restraints
H atoms treated by a mixture of independent and constrained refinement
Δρ_max_ = 0.28 e Å^−3^
Δρ_min_ = −0.28 e Å^−3^

### 

Data collection: *APEX2* (Bruker, 2007 ▶); cell refinement: *SAINT* (Bruker, 2007 ▶); data reduction: *SAINT*; program(s) used to solve structure: *SIR97* (Altomare *et al.*, 1999 ▶); program(s) used to refine structure: *SHELXL97* (Sheldrick, 2008 ▶); molecular graphics: *ORTEP-3 for Windows* (Farrugia, 1997 ▶); software used to prepare material for publication: *WinGX* (Farrugia, 1999 ▶) and *PLATON* (Spek, 2009 ▶).

## Supplementary Material

Crystal structure: contains datablock(s) global, I. DOI: 10.1107/S1600536811055528/xu5426sup1.cif

Structure factors: contains datablock(s) I. DOI: 10.1107/S1600536811055528/xu5426Isup2.hkl

Supplementary material file. DOI: 10.1107/S1600536811055528/xu5426Isup3.cml

Additional supplementary materials:  crystallographic information; 3D view; checkCIF report

## Figures and Tables

**Table 1:** Hydrogen-bond geometry (Å, °)

*D*—H⋯*A*	*D*—H	H⋯*A*	*D*⋯*A*	*D*—H⋯*A*
N1—H1*N*⋯O3^i^	0.86 (2)	2.07 (2)	2.904 (3)	165 (2)
N2—H2*N*⋯O2^ii^	0.85 (2)	2.25 (2)	3.075 (3)	164 (2)
C9—H9⋯O1^iii^	0.93	2.59	3.308 (3)	135

Symmetry codes: (i) 
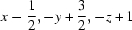
; (ii) 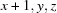; (iii) 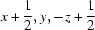.

**Fig. 1.**
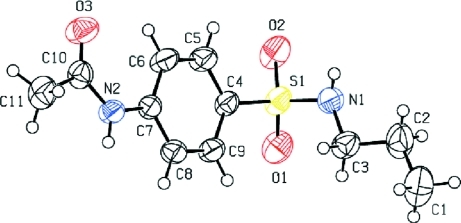
View of the title compound (I) showing the atom-labelling scheme and displacement ellipsoids at the 50% probability level.

**Fig. 2.**
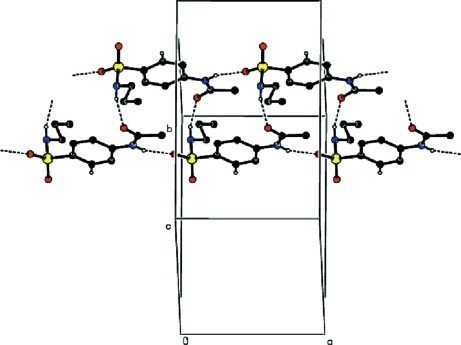
View of the molecules linked by N—H···O hydrogen bonds as the R32(18) ring motifs. H atoms not involved in hydrogen bonds (dashed lines) and C—H···O intreractions have been omitted for clarity.

## supplementary crystallographic information

### Comment

Sulfonamides are derivatives of *p*-aminobenzene sulfonic acid (Ahrens, 1996) belong to the oldest group of antibiotics which are also being used now-a-days. These are white crystalline powder derived from azo dye (Adams, 2001) and have weak organic acid characteristics with a structural resemblance to *p*-aminobenzoic acid which is an intermediate required for the synthesis of folic acid in bacteria (Ahrens, 1996). The sensitivity of sulfonamides is dependent on the mode in which organisms fulfill their folic acid requirements. Sulfonamides are considered as bacteriostatic drugs (Mayers, 2009 & Betts *et al.*, 2003) which are used for the treatment of systematic infections and are absorbed in the gastrointestinal tract (Root, 1999).

As part of our ongoing studies (Faryal *et al.*, 2011, Ahmad *et al.* (2011*a*,*b*), we synthesized the title compound, (I), and report herein its crystal structure.

In the title compound, (Fig. 1), the dihedral angles between the benzene ring (C4—C9) and the propylsulfonamide (C1,C2,C3,N1,S1) and methylamide (N2,C10,O3,C11) moieties are 71.8 (1) and 5.8 (1)°, respectively. The S atom has a distorted tetrahedral geometry [maximum deviation: O—S—O = 119.48 (15)°]. The C—S—N—C torsion angles are 66.1 (2)°.

In the crystal, the molecules are linked by N_m_—H···O_s_ (m = methylamide, s = sulfonamide) hydrogen bonds, forming C(8) chains along the *a* axis (Table1, Fig. 2). The two molecule chains also connect by N—H···O hydrogen bonds, generating *R*_3_^2^(18) rings (Bernstein *et al.*, 1995; Etter *et al.*, 1990; Motherwell *et al.*, 1999; Table 1, Fig. 2). The crystal packing is further stabilized by the intermolecular C—H···O hydrogen bonds.

### Experimental

10 m*M* of 4-acetamido benzene sulfonyl chloride was taken in the reaction flask and about 20 ml distilled water was added in it. Mixed it well. Then 10 m*M* of propylamine hydrochloride was added in it. 3% Na_2_CO_3_ was used to maintain the pH at 8–10. The reaction was stirred for about 2 h to get the maximum yield. Precipitates obtained was filtered and dried. They are recrystallized in the mixture of methanol and ethyl acetate 1:1. The reaction was monitored by TLC.

### Refinement

The N-bound H atoms were located in difference Fourier maps and isotropically refined with the N–H distance restraint [0.86 (1) Å)]. The C-bound H atoms were geometrically placed using a riding model with C—H = 0.93 - 0.97 Å, and *U*_iso_(H)= 1.2*U*_eq_(C_aromatic_, C_methylene_) and *U*_iso_(H) = 1.5*U*_eq_(C_methyl_).

### Crystal data

**Table:**

C_11_H_16_N_2_O_3_S	*F*(000) = 1088
*M**_r_* = 256.33	*D*_x_ = 1.357 Mg m^−^^3^
Orthorhombic, *P**b**c**a*	Mo *K*α radiation, λ = 0.71073 Å
Hall symbol: -P 2ac 2ab	Cell parameters from 1934 reflections
*a* = 8.7791 (6) Å	θ = 2.9–21.9°
*b* = 14.1747 (11) Å	µ = 0.26 mm^−^^1^
*c* = 20.1577 (14) Å	*T* = 296 K
*V* = 2508.5 (3) Å^3^	Block, colourless
*Z* = 8	0.13 × 0.12 × 0.10 mm

### Data collection

**Table:**

Bruker APEXII CCD diffractometer	1579 reflections with *I* > 2σ(*I*)
Radiation source: sealed tube	*R*_int_ = 0.077
graphite	θ_max_ = 28.3°, θ_min_ = 2.0°
φ and ω scans	*h* = −10→11
22013 measured reflections	*k* = −18→18
3103 independent reflections	*l* = −26→23

### Refinement

**Table:**

Refinement on *F*^2^	Primary atom site location: structure-invariant direct methods
Least-squares matrix: full	Secondary atom site location: difference Fourier map
*R*[*F*^2^ > 2σ(*F*^2^)] = 0.057	Hydrogen site location: inferred from neighbouring sites
*wR*(*F*^2^) = 0.173	H atoms treated by a mixture of independent and constrained refinement
*S* = 1.01	*w* = 1/[σ^2^(*F*_o_^2^) + (0.0795*P*)^2^ + 0.2926*P*] where *P* = (*F*_o_^2^ + 2*F*_c_^2^)/3
3103 reflections	(Δ/σ)_max_ < 0.001
164 parameters	Δρ_max_ = 0.28 e Å^−^^3^
2 restraints	Δρ_min_ = −0.28 e Å^−^^3^

### Special details

**Table:**

Geometry. Bond distances, angles *etc*. have been calculated using the rounded fractional coordinates. All su's are estimated from the variances of the (full) variance-covariance matrix. The cell e.s.d.'s are taken into account in the estimation of distances, angles and torsion angles
Refinement. Refinement on *F*^2^ for ALL reflections except those flagged by the user for potential systematic errors. Weighted *R*-factors *wR* and all goodnesses of fit *S* are based on *F*^2^, conventional *R*-factors *R* are based on *F*, with *F* set to zero for negative *F*^2^. The observed criterion of *F*^2^ > σ(*F*^2^) is used only for calculating -*R*-factor-obs *etc*. and is not relevant to the choice of reflections for refinement. *R*-factors based on *F*^2^ are statistically about twice as large as those based on *F*, and *R*-factors based on ALL data will be even larger.

### Fractional atomic coordinates and isotropic or equivalent isotropic displacement parameters (Å^2^)

**Table:**

	*x*	*y*	*z*	*U*_iso_*/*U*_eq_	
S1	0.08384 (8)	0.62424 (6)	0.34117 (4)	0.0520 (3)	
O1	0.0871 (3)	0.55742 (16)	0.28801 (9)	0.0662 (8)	
O2	−0.0349 (2)	0.61902 (17)	0.38968 (11)	0.0702 (9)	
O3	0.6209 (3)	0.63450 (16)	0.58686 (10)	0.0681 (9)	
N1	0.0734 (3)	0.7275 (2)	0.30872 (12)	0.0530 (9)	
N2	0.6813 (3)	0.61300 (18)	0.47900 (11)	0.0492 (9)	
C1	0.2273 (4)	0.8744 (3)	0.1670 (2)	0.0893 (16)	
C2	0.1289 (4)	0.8443 (3)	0.22353 (19)	0.0803 (16)	
C3	0.1785 (3)	0.7554 (2)	0.25581 (13)	0.0557 (10)	
C4	0.2588 (3)	0.61648 (19)	0.38364 (13)	0.0431 (10)	
C5	0.2664 (3)	0.6361 (2)	0.45047 (14)	0.0532 (10)	
C6	0.4031 (3)	0.6343 (2)	0.48376 (14)	0.0546 (10)	
C7	0.5362 (3)	0.61413 (19)	0.44927 (13)	0.0419 (9)	
C8	0.5275 (3)	0.5935 (2)	0.38184 (13)	0.0474 (10)	
C9	0.3906 (3)	0.5945 (2)	0.34959 (13)	0.0492 (10)	
C10	0.7159 (4)	0.6219 (2)	0.54421 (15)	0.0494 (11)	
C11	0.8829 (3)	0.6153 (2)	0.56009 (17)	0.0667 (14)	
H1A	0.32730	0.88930	0.18310	0.1340*	
H1B	0.18390	0.92900	0.14620	0.1340*	
H1C	0.23410	0.82410	0.13520	0.1340*	
H1N	0.071 (3)	0.7722 (14)	0.3372 (10)	0.047 (9)*	
H2A	0.12780	0.89410	0.25650	0.0960*	
H2B	0.02550	0.83630	0.20750	0.0960*	
H2N	0.754 (2)	0.604 (2)	0.4517 (12)	0.060 (10)*	
H3A	0.18360	0.70560	0.22290	0.0670*	
H3B	0.27970	0.76380	0.27420	0.0670*	
H5	0.17760	0.65070	0.47350	0.0640*	
H6	0.40650	0.64660	0.52910	0.0660*	
H8	0.61580	0.57900	0.35850	0.0570*	
H9	0.38620	0.58020	0.30460	0.0590*	
H11A	0.92890	0.67640	0.55550	0.1000*	
H11B	0.93090	0.57180	0.53010	0.1000*	
H11C	0.89560	0.59340	0.60480	0.1000*	

### Atomic displacement parameters (Å^2^)

**Table:**

	*U*^11^	*U*^22^	*U*^33^	*U*^12^	*U*^13^	*U*^23^
S1	0.0345 (4)	0.0769 (6)	0.0447 (4)	−0.0061 (4)	−0.0052 (3)	0.0085 (4)
O1	0.0641 (15)	0.0778 (16)	0.0568 (12)	−0.0094 (12)	−0.0178 (11)	−0.0047 (12)
O2	0.0343 (12)	0.116 (2)	0.0604 (13)	−0.0096 (12)	0.0010 (10)	0.0234 (13)
O3	0.0522 (14)	0.1056 (19)	0.0465 (12)	−0.0005 (12)	−0.0017 (11)	−0.0090 (12)
N1	0.0418 (15)	0.0712 (19)	0.0461 (14)	0.0069 (13)	−0.0014 (12)	0.0017 (13)
N2	0.0309 (14)	0.0773 (18)	0.0395 (13)	0.0056 (12)	0.0018 (11)	0.0019 (12)
C1	0.051 (2)	0.117 (3)	0.100 (3)	−0.003 (2)	0.003 (2)	0.049 (2)
C2	0.055 (2)	0.103 (3)	0.083 (3)	0.014 (2)	0.008 (2)	0.037 (2)
C3	0.0451 (18)	0.075 (2)	0.0471 (16)	−0.0035 (17)	0.0025 (14)	0.0057 (16)
C4	0.0339 (16)	0.0585 (19)	0.0370 (14)	−0.0018 (13)	−0.0012 (11)	0.0061 (13)
C5	0.0311 (16)	0.085 (2)	0.0436 (16)	0.0025 (15)	0.0065 (13)	0.0038 (15)
C6	0.0385 (17)	0.092 (2)	0.0334 (14)	0.0001 (16)	0.0019 (13)	0.0000 (14)
C7	0.0328 (15)	0.0541 (18)	0.0388 (14)	0.0023 (13)	0.0016 (12)	0.0059 (13)
C8	0.0353 (16)	0.063 (2)	0.0438 (16)	0.0039 (14)	0.0058 (12)	0.0025 (14)
C9	0.0422 (18)	0.069 (2)	0.0363 (15)	0.0029 (14)	0.0004 (13)	−0.0001 (14)
C10	0.0410 (18)	0.056 (2)	0.0512 (18)	−0.0010 (15)	−0.0058 (14)	0.0038 (14)
C11	0.043 (2)	0.092 (3)	0.065 (2)	−0.0018 (17)	−0.0138 (16)	0.0075 (18)

### Geometric parameters (Å, °)

**Table:**

S1—O1	1.431 (2)	C8—C9	1.367 (4)
S1—O2	1.431 (2)	C10—C11	1.504 (4)
S1—N1	1.606 (3)	C1—H1A	0.9600
S1—C4	1.762 (3)	C1—H1B	0.9600
O3—C10	1.211 (4)	C1—H1C	0.9600
N1—C3	1.465 (4)	C2—H2A	0.9700
N2—C7	1.408 (4)	C2—H2B	0.9700
N2—C10	1.355 (4)	C3—H3A	0.9700
N1—H1N	0.86 (2)	C3—H3B	0.9700
N2—H2N	0.85 (2)	C5—H5	0.9300
C1—C2	1.492 (5)	C6—H6	0.9300
C2—C3	1.484 (5)	C8—H8	0.9300
C4—C9	1.381 (4)	C9—H9	0.9300
C4—C5	1.377 (4)	C11—H11A	0.9600
C5—C6	1.375 (4)	C11—H11B	0.9600
C6—C7	1.389 (4)	C11—H11C	0.9600
C7—C8	1.392 (4)		
			
O1—S1—O2	119.48 (15)	C2—C1—H1C	109.00
O1—S1—N1	107.43 (13)	H1A—C1—H1B	109.00
O1—S1—C4	107.77 (14)	H1A—C1—H1C	109.00
O2—S1—N1	106.49 (14)	H1B—C1—H1C	109.00
O2—S1—C4	107.44 (13)	C1—C2—H2A	109.00
N1—S1—C4	107.73 (13)	C1—C2—H2B	109.00
S1—N1—C3	120.5 (2)	C3—C2—H2A	109.00
C7—N2—C10	127.9 (3)	C3—C2—H2B	109.00
S1—N1—H1N	113.8 (13)	H2A—C2—H2B	108.00
C3—N1—H1N	107.7 (16)	N1—C3—H3A	109.00
C7—N2—H2N	113.9 (15)	N1—C3—H3B	109.00
C10—N2—H2N	118.2 (15)	C2—C3—H3A	109.00
C1—C2—C3	114.1 (3)	C2—C3—H3B	109.00
N1—C3—C2	111.3 (2)	H3A—C3—H3B	108.00
C5—C4—C9	119.4 (2)	C4—C5—H5	119.00
S1—C4—C5	120.3 (2)	C6—C5—H5	119.00
S1—C4—C9	120.2 (2)	C5—C6—H6	120.00
C4—C5—C6	121.1 (3)	C7—C6—H6	120.00
C5—C6—C7	119.6 (3)	C7—C8—H8	120.00
N2—C7—C6	123.4 (2)	C9—C8—H8	120.00
N2—C7—C8	117.6 (2)	C4—C9—H9	120.00
C6—C7—C8	119.1 (2)	C8—C9—H9	120.00
C7—C8—C9	120.7 (2)	C10—C11—H11A	109.00
C4—C9—C8	120.2 (2)	C10—C11—H11B	109.00
O3—C10—C11	122.0 (3)	C10—C11—H11C	109.00
N2—C10—C11	114.8 (3)	H11A—C11—H11B	109.00
O3—C10—N2	123.2 (3)	H11A—C11—H11C	109.00
C2—C1—H1A	110.00	H11B—C11—H11C	109.00
C2—C1—H1B	110.00		
			
O1—S1—N1—C3	−49.8 (3)	C7—N2—C10—O3	−1.6 (5)
O2—S1—N1—C3	−178.9 (2)	C1—C2—C3—N1	−177.3 (3)
C4—S1—N1—C3	66.1 (2)	S1—C4—C5—C6	−177.2 (2)
N1—S1—C4—C9	−82.9 (3)	S1—C4—C9—C8	176.5 (2)
N1—S1—C4—C5	94.4 (2)	C5—C4—C9—C8	−0.9 (4)
O1—S1—C4—C5	−149.9 (2)	C9—C4—C5—C6	0.1 (4)
O2—S1—C4—C5	−20.0 (3)	C4—C5—C6—C7	1.2 (4)
O1—S1—C4—C9	32.8 (3)	C5—C6—C7—N2	178.4 (3)
O2—S1—C4—C9	162.8 (2)	C5—C6—C7—C8	−1.8 (4)
S1—N1—C3—C2	167.9 (2)	N2—C7—C8—C9	−179.2 (3)
C10—N2—C7—C6	6.5 (5)	C6—C7—C8—C9	1.1 (4)
C7—N2—C10—C11	178.6 (3)	C7—C8—C9—C4	0.3 (4)
C10—N2—C7—C8	−173.2 (3)		

### Hydrogen-bond geometry (Å, °)

**Table:**

*D*—H···*A*	*D*—H	H···*A*	*D*···*A*	*D*—H···*A*
N1—H1N···O3^i^	0.86 (2)	2.07 (2)	2.904 (3)	165 (2)
N2—H2N···O2^ii^	0.85 (2)	2.25 (2)	3.075 (3)	164 (2)
C9—H9···O1^iii^	0.93	2.59	3.308 (3)	135

Symmetry codes: (i) *x*−1/2, −*y*+3/2, −*z*+1; (ii) *x*+1, *y*, *z*; (iii) *x*+1/2, *y*, −*z*+1/2.
